# Tree Nuts Are Inversely Associated with Metabolic Syndrome and Obesity: The Adventist Health Study-2

**DOI:** 10.1371/journal.pone.0085133

**Published:** 2014-01-08

**Authors:** Karen Jaceldo-Siegl, Ella Haddad, Keiji Oda, Gary E. Fraser, Joan Sabaté

**Affiliations:** 1 Department of Nutrition, School of Public Health, Loma Linda University, Loma Linda, California, United States of America; 2 Department of Epidemiology & Biostatistics, School of Public Health, Loma Linda University, Loma Linda, California, United States of America; 3 Adventist Health Studies, School of Public Health, Loma Linda University, Loma Linda, California, United States of America; University College London, United Kingdom

## Abstract

**Objective:**

To examine the relationships of nut consumption, metabolic syndrome (MetS), and obesity in the Adventist Health Study-2, a relatively healthy population with a wide range of nut intake.

**Research Design and Methods:**

Cross-sectional analysis was conducted on clinical, dietary, anthropometric, and demographic data of 803 adults. MetS was defined according to the American Heart Association and the National Heart, Lung, and Blood Institute diagnostic criteria. We assessed intake of total nuts, tree nuts and peanuts, and also classified subjects into low tree nut/low peanut (LT/LP), low tree/high peanut (LT/HP), high tree nut/high peanut (HT/HP), and high tree/low peanut (HT/LP) consumers. Odds ratios were estimated using multivariable logistic regression.

**Results:**

32% of subjects had MetS. Compared to LT/LP consumers, obesity was lower in LT/HP (OR = 0.89; 95% CI = 0.53, 1.48), HT/HP (OR = 0.63; 95% CI = 0.40, 0.99) and HT/LP (OR = 0.54; 95% CI = 0.34, 0.88) consumers, *p* for trend = 0.006. For MetS, odds ratios (95% CI) were 0.77 (0.47, 1.28), 0.65 (0.42, 1.00) and 0.68 (0.43, 1.07), respectively (*p* for trend = 0.056). Frequency of nut intake (once/week) had significant inverse associations with MetS (3% less for tree nuts and 2% less for total nuts) and obesity (7% less for tree nuts and 3% less for total nuts).

**Conclusions:**

Tree nuts appear to have strong inverse association with obesity, and favorable though weaker association with MetS independent of demographic, lifestyle and dietary factors.

## Introduction

Metabolic syndrome (MetS) is a cluster of multiple metabolic risk factors shown to be associated with death, a twofold increased risk for cardiovascular disease, and a fivefold increased risk for type 2 diabetes [1], [2], [3]. Diagnostic criteria for MetS vary, but the main features include abdominal obesity, elevated triglycerides (TG), reduced HDL-C, elevated blood pressure (BP), and hyperglycemia. Presence of any three of these five conditions constitutes a diagnosis of MetS according to the American Heart Association and the National Heart, Lung, and Blood Institute (AHA/NHLBI) [4]. Between 20% and 30% of the adult population worldwide can be characterized as having MetS [5], and in the United States (US), the prevalence is estimated at 34.3%, based on NHANES data from 2003–2006 [6]. Because MetS is a major risk factor for cardiovascular disease and *type 2 diabetes*, preventing or reversing MetS is of paramount importance.

Nut consumption has been found to improve blood lipid levels [7] and reduce the risk of coronary heart disease [8], [9]. Nuts are energy-dense foods high in total fat (50–75% by weight) thus perceived as fattening. Since obesity has become a major public health problem and is a risk factor for cardiovascular disease, it is very pertinent to determine if nut consumption increases the risk of obesity. Few epidemiologic studies have assessed the association between nut intake and BMI or the risk of obesity. We have previously reported an inverse relationship between nut consumption and BMI in the Adventist Health Study 1 cohort [10], but no association was found in the Physician's Health Study [11]. In the Nurses' Health Study II, participants who consumed nuts frequently (two or more times per week) had a 31% reduced risk of weight gain, or a 33% lower risk of obesity [12] than those who rarely or never consumed nuts. Also, in short-term dietary intervention studies, nuts do not appear to contribute to weight gain [12], [13], [14]. Results from a recent meta-analysis of clinical trials conclude that nut-enriched diets do not increase body weight, BMI or waist circumference [15].

Although still limited, the number of publications on nut intake and MetS is increasing. Results are challenging to translate in part due to variations in the assessment or definition of nut consumption. For example, out-of-hand nut intake (peanuts + tree nuts) was cross-sectionally associated with a lower prevalence of two risk factors for MetS, but not MetS in adults from the NHANES 1999–2004 study cohort [16]. When subjects from the same cohort were classified as consumers or non-consumers of tree nuts or total nuts, tree nut consumers compared to non-consumers had a lower prevalence of four risk factors of MetS as well as MetS [17]. In prospective studies, nut consumption (tree nuts + peanuts) of two or more servings per week was associated with lower incidence of MetS [18], but no association was observed with a prudent dietary pattern which includes nuts [19]. In the PREDIMED (Prevención con Dieta Mediterránea) trial, a reversal of MetS was observed with a Mediterranean diet enhanced with mixed tree nuts after one year of follow-up [20].

In the present study, we sought to investigate the relationship between nut consumption and MetS in the Adventist Health Study-2 (AHS-2) cohort, a relatively healthy population geographically spread throughout the US and Canada with a wide range of nut intake ranging from never to once or more daily. The intake of nuts varies greatly in the population due to personal preferences. In an attempt to capture this variation, we assessed nut intake using different combinations: tree nuts and peanuts separately, the combination of both tree nuts and peanuts in varying amounts, and total nuts, and hypothesized that the consumption of tree nuts is associated with lower rates of MetS and obesity.

## Materials and Methods

### Ethics Statement

The Institutional Review Board of Loma Linda University approved the study protocol, and all study participants provided written consent at the time of enrollment. Study design and subject selection

We conducted cross-sectional analysis on data from the AHS-2 Calibration Sub-study, a representative sample of the AHS-2 cohort. Recruitment and selection methods of the parent cohort have been described previously [21]. Briefly, adult members (age 30+ years) of Seventh-day Adventist churches throughout the US and Canada were enrolled and completed the baseline AHS-2 “Connecting Lifestyle to Disease and Longevity” Questionnaire, which included medical history, dietary habits, physical activity, and demographic information. Approximately 27% of the cohort is black of US and Caribbean origin and the remaining participants are primarily white with a minor proportion from other races. The Calibration Sub-study participants (n = 1011) represent the parent cohort by gender, age, and education [22]. Participants were randomly selected from the AHS-2 cohort by church and then within the church by gender and age. Because of the special interest in black Adventists, we oversampled (45%) this minority population. Calibration study participants attended a clinic at their local church during which waist circumference, height, weight, and blood pressure were measured, and fasting blood samples were collected. The analytic sample included individuals who had complete data on all relevant variables (n = 803).

### Dietary assessment

Dietary intake was assessed by a self-administered food frequency questionnaire (FFQ) at baseline, which contains a list of over 200 foods including eight items on nuts. The FFQ was validated against multiple 24-hour dietary recalls. In general, validity correlations were moderate to high for macronutrients, fatty acids, vitamins, minerals, and fiber [22]. Validity correlations for tree nuts, peanuts, peanut butter and total nuts in whites were 0.58, 0.40, 0.59, and 0.58, respectively. In blacks, correlations were 0.39, 0.27, 0.41, and 0.47, respectively [23]. For the current analysis, we assessed separately the intake of tree nuts (T), peanuts + peanut butter (P) and total nuts (T + P) in terms of the amount and frequency of nuts consumed. We also classified subjects by the type of nuts consumed: low tree nuts/low peanuts (LT/LP), low tree nuts/high peanuts (LT/HP), high tree nuts/high peanuts (HT/HP), and high tree nuts/low peanuts (HT/LP). Nutrient composition of foods was based on the Nutrition Data System for Research 2008 database (NDS-R, Nutrition Coordinating Center, Minneapolis, MN, USA). Subjects with estimated total energy intake of <500 kcal or >4500 kcal were excluded from the analyses.

### Diagnostic criteria for metabolic syndrome

MetS was defined according to the AHA/NHLBI diagnostic criteria [4]. Individuals with three of the following five conditions are characterized as having MetS: abdominal obesity [waist circumference ≥102 cm (≥ 40 inches) in men, ≥ 88 cm (≥ 35 inches) in women]; hypertriglyceridemia [TG≥150 mg/dL (≥1.7 mmol/L) or drug treatment for elevated TG]; low HDL-C [< 40 mg/dL (< 1.03 mmol/L) in men, <50 mg/dL (< 1.3 mmol/L) in women, or drug treatment of reduced HDL-C]; elevated BP [≥130 mm Hg systolic BP, or ≥85 mm Hg diastolic BP, or drug treatment of hypertension]; hyperglycemia [fasting plasma glucose ≥100 mg/dL or drug treatment for elevated glucose]. Information about medication use was elicited by questionnaire at baseline and through interviews during the Calibration Study.

Fasting plasma glucose, HDL-C, and TG concentrations were obtained via finger stick using the Cholestech LDX System (Cholestech, Hayward, CA). Validity of the finger stick against venous samples has been reported previously [24]. Blood pressure was assessed as the average of three measurements using the Omron Automatic Digital Blood Pressure Monitor HEM-747IC (Omron Healthcare, Inc., Vernon Hills, IL). Waist circumference was measured with an anthropometric tape. Three measurements were taken, and the mean value was used in the analysis. Height was measured to the nearest quarter inch (0.64 cm) using the Seca 214 Portable Height Rod (Seca Corp., Hamburg, Germany), and weight to the nearest half pound (0.23 kg) using Tanita BF-350 (Tanita UK Ltd., Middlesex, UK). Body mass index (BMI) was calculated as weight divided by height squared (kg/m^2^). Obesity was defined as BMI ≥30 kg/m^2^ [25].

### Lifestyle and sociodemographic factors

Sociodemographic and lifestyle factors assessed by questionnaire at baseline included age, gender, ethnicity (white or black), education (≤high school, some college, ≥Bachelor's degree), cigarette use (ever or never), and alcohol use (ever or never). Sedentary time was quantified as hours per day spent in physical inactivity, such as watching television or reading while lying down. Other factors associated with the MetS include an inverse relationship with whole grains [26], [27] and dairy [28], while meat intake has been shown to be associated with MetS [19] and adiposity measures [29].

### Statistical analysis

To produce four balanced categories of nut intake, we used 2 times/week as cutoff points for both tree nut and peanut variables. Means (SD) or proportions were calculated for all relevant variables. Differences according to type of nuts consumed were determined using Chi-square test for categorical and one-way ANOVA or Kruskal-Wallis (for variables not normally distributed) test for continuous variables. Odds ratios (95% CI) were calculated using multivariable logistic regression to determine associations of nut intake pattern (low tree nut/low peanut, low tree nut/high peanut, high tree nut/high peanut, and high tree nut/low peanut with low tree nut/low peanut as reference) with obesity, each component of MetS (abdominal obesity, hypertriglyceridemia, low HDL-C, high blood pressure, and hyperglycemia), and MetS. Regression analyses were adjusted for age, gender, ethnicity, education, cigarette use, alcohol use, hours of sedentary time per day, and energy, red meat, whole grain and dairy intake. We also quantified each type of nut (tree nuts, peanuts, and total nuts) according to frequency (1 time/week as the unit) and amount (1 serving/week as the unit) of nuts consumed, and using multivariable logistic regression, evaluated their relationship with MetS and obesity adjusting for the same demographic and lifestyle factors. However, we additionally adjusted for peanuts when tree nut intake was tested as the main effect, and adjusted for tree nuts when peanuts were the primary independent variable.

## Results

Mean tree nut intake was 16 g/d among high tree nut consumers, and 5 g/d among low tree nut consumers. Low and high peanut consumers had mean peanut intake of 4 g/d and 14 g/d, respectively. High tree nut compared to low tree nut consumers were significantly older, white, never smoked, never used alcohol, had less sedentary time, had lower body weight, BMI and percent body fat, and consumed more energy/day (Table 1). Intake of total carbohydrates, plant protein, saturated (SFA), monounsaturated (MUFA) and polyunsaturated (PUFA) fatty acids, total fiber, α-tocopherol, and magnesium was generally lowest among the lowest nut consumers (LT/LP), highest among the highest nut consumers (HT/HP), and intermediate among the other two groups (LT/HP and HT/LP) (all with *p*<0.0001). However, intake of animal protein (*p*<0.0001) and trans-fatty acid (TFA) (*p*<0.0001) was lower among high tree nut consumers compared to low tree nut consumers (Table 1).

**Table 1:** Unadjusted means and proportions of selected participant characteristics according to type of nut intake.

	Low Tree Nut		High Tree Nut		
	Low peanut	High peanut	High peanut	Low peanut	*p*-value
n (%)	284 (35.4)	110 (13.7)	242 (30.1)	167 (20.8)	
*Nut intake*					
Tree nut in g/d, mean (SD)	4.9 (3.6)	3.2 (3.3)	16.8 (15.4)	16.3 (17.4)	<0.0001*
Peanut in g/d, mean (SD)	3.6 (2.5)	14.3 (10.3)	14.8 (11.7)	2.9 (2.5)	<0.0001*
Total nut in g/d, mean (SD)	8.5 (6.0)	16.5 (11.5)	31.6 (22.3)	18.8 (16.7)	<0.0001*
*Demographic factors*					
Age in years, mean (SD)	53.3 (12.9)	56.4 (12.5)	60.4 (13.1)	60.1 (12.6)	<0.0001†
Gender					0.017‡
Females, n (%)	189 (66.6)	63 (57.3)	144 (59.5)	121 (72.5)	
Males, n (%)	95 (33.4)	47 (42.7)	98 (40.5)	46 (27.5)	
Ethnicity					<0.0001‡
Whites, n (%)	116 (40.9)	70 (63.6)	171 (70.7)	105 (62.9)	
Blacks, n (%)	168 (59.1)	40 (36.4)	71 (29.3)	62 (37.1)	
Education					0.25‡
Less than college, n (%)	62 (21.8)	24 (21.8)	41 (16.9)	32 (19.2)	
Some college, n (%)	123 (43.4)	45 (40.9)	93 (38.4)	60 (35.9)	
College grad+, n (%)	99 (34.9)	41 (37.3)	108 (44.6)	75 (44.9)	
*Lifestyle factors*					
Smoking					0.015‡
Never, n (%)	220 (77.5)	93 (84.5)	212 (87.6)	142 (85.0)	
Ever, n (%)	64 (22.5)	17 (15.5)	30 (12.4)	25 (15.0)	
Alcohol use					<0.0001‡
Never, n (%)	136 (47.9)	61 (55.5)	163 (67.4)	112 (67.1)	
Ever, n (%)	148 (52.1)	49 (44.5)	79 (32.6)	55 (32.9)	
Sedentary time in hour/day, mean (SD)	0.97 (1.28)	0.79 (1.06)	0.62 (0.96)	0.71 (1.06)	0.0007*
Energy in kcals, mean (SD)	1634.9 (729.1)	1982.9 (737.6)	2342.7 (795.0)	1913.3 (724.5)	<0.0001*
*Metabolic risk factors*					
Body weight in kg, mean (SD)	82.1 (21.1)	82.5 (20.1)	76.4 (18.6)	73.3 (17.3)	<0.0001*
BMI, mean (SD)	29.8 (7.7)	28.7 (6.4)	27.2 (5.9)	26.6 (6.1)	<0.0001*
Percent body fat, mean (SD)	36.1 (10.7)	34.5 (10.6)	32.9 (9.5)	33.2 (9.5)	0.0021†
Waist circumference in cm, mean (SD)	95.6 (17.7)	97.3 (15.3)	92.9 (15.3)	89.9 (15.6)	0.0002*
Triglycerides in mg/dL, mean (SD)	123.4 (81.4)	120.6 (69.1)	124.3 (68.5)	120.2 (62.8)	0.91*
HDL cholesterol in mg/dL, mean (SD)	49.4 (14.2)	47.0 (13.9)	48.8 (15.6)	49.7 (14.9)	0.61*
Systolic BP in mm/Hg, mean (SD)	124.7 (20.4)	126.4 (19.6)	128.8 (21.1)	126.4 (19.1)	0.15†
Diastolic BP in mm/Hg, mean (SD)	78.1 (10.5)	76.9 (9.5)	76.7 (9.8)	76.4 (10.3)	0.30†
Glucose in mg/dL, mean (SD)	95.9 (26.0)	100.6 (37.6)	93.8 (23.5)	91.8 (20.3)	0.12*
*Nutrient intake, mean (SD)*					
Energy (kcal/d)	1635 (729)	1983 (738)	2343 (795)	1913 (725)	<0.0001*
Carbohydrate (%EI)	54.4 (10.8)	54.4 (10.8)	51.7 (69.1)	55.8 (10.0)	0.0002†
Total protein (%EI)	14.0 (3.5)	13.9 (2.3)	14.2 (2.7)	13.9 (2.8)	0.63*
Plant protein (%EI)	9.1 (3.2)	9.8 (2.7)	10.7 (2.7)	10.8 (3.4)	<0.0001*
Animal protein (%EI)	4.9 (3.5)	4.1 (2.8)	3.5 (3.1)	3.1 (2.4)	<0.0001*
Total fat (%EI)	29.7 (9.0)	32.3 (7.9)	34.0 (7.7)	31.6 (8.4)	<0.0001*
SFA (%EI)	7.4 (2.8)	8.0 (3.0)	7.3 (2.3)	6.9 (2.9)	0.003*
MUFA (%EI)	11.9 (4.4)	13.0 (3.8)	14.6 (4.4)	13.1 (4.4)	<0.0001*
PUFA (%EI)	8.3 (2.7)	8.9 (1.9)	9.8 (2.2)	9.3 (2.6)	<0.0001*
TFA (%EI)	1.9 (1.4)	2.0 (1.0)	1.5 (0.9)	1.4 (1.0)	<0.0001*
Total fiber (%FW)	1.0 (0.4)	1.1 (0.4)	1.3 (0.4)	1.2 (0.4)	<0.0001*
Insoluble fiber (%FW)	0.7 (0.3)	0.7 (0.3)	0.9 (0.3)	0.8 (0.3)	<0.0001*
Soluble fiber (%FW)	0.3 (0.1)	0.3 (0.1)	0.4 (0.1)	0.4 (0.1)	<0.0001*
α-Tocopherol dietary (μ/d)	7.2 (3.3)	9.8 (3.9)	15.0 (6.7)	11.8 (6.7)	<0.0001*
α-Tocopherol total (μ/d)	63.7 (114.1)	76.9 (129.0)	110.3 (137.6)	110.3 (148.8)	<0.0001*
Magnesium (mg/d)	350.5 (176.7)	452.7 (215.9)	610.0 (239.2)	525.3 (265.3)	<0.0001*

Kruskal-Wallis test was used in the analysis of non-normally distributed variables.

One-way ANOVA was used in the analysis.

Chi-square test was used in the analysis.

**Figure 1:**
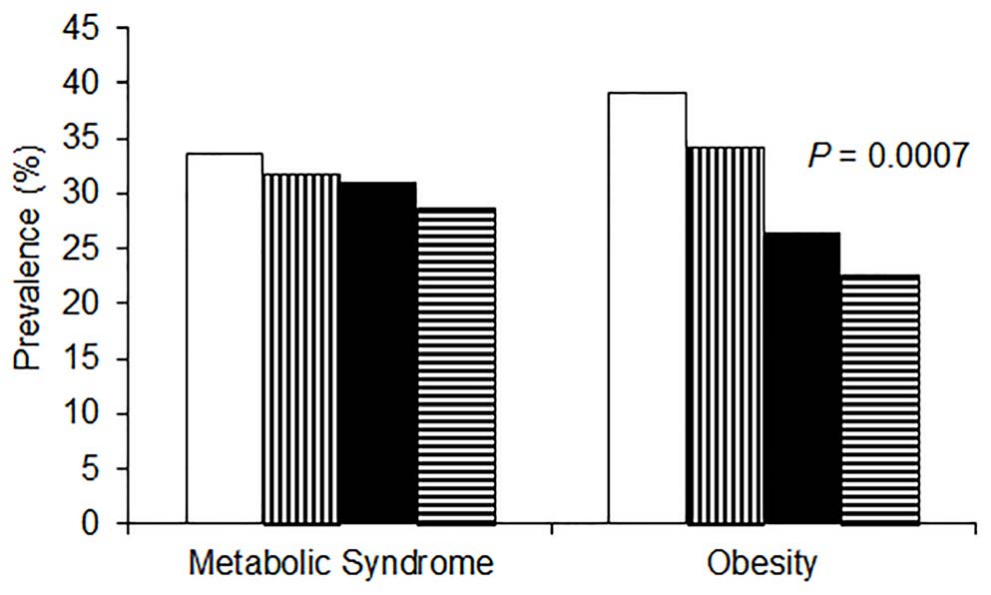
Prevalence (%) of metabolic syndrome and obesity according to type of nuts consumed. Metabolic syndrome was defined according to the AHS/NHLBI diagnostic criteria [4]; obesity: BMI ≥30 kg/m^2^ [22]. Chi-square test was used to determine differences in prevalence by type of nuts consumed: no fill (low tree nut/low peanut), vertical (low tree nut/high peanut), black fill (high tree nut/high peanut), horizontal (high tree nut/low peanut).

In all, 32% of subjects had MetS. Prevalence of MetS in LT/LP, LT/HP, HT/HP and HT/LP, was 33.5%, 31.8%, 31.0%, and 28.7%, respectively (Figure 1). High tree nut consumers (HT/LP and HT/HP) compared to low tree nut consumers (LT/LP, LT/HP) significantly had lower prevalence of obesity (*p* = 0.0007).

In multivariable analysis (Table 2), abdominal obesity was significantly lower in HT/LP than in LT/LP consumers (OR = 0.60; 95% CI 0.39–0.93, *p* for trend = 0.02). Compared to LT/LP consumers, hypertriglyceridemia tended to decrease with increased tree nut consumption (with the lowest values among the high tree nut/low peanut consumers), and this association nearly reached significance. Hyperglycemia, high blood pressure, or low HDL-C was not associated with type of nuts consumed.

**Table 2:** Multivariable-adjusted odds ratios (95% confidence interval) relating obesity and metabolic syndrome risk factors according to type of nut intake.

	Low Tree Nut		High Tree Nut		
Metabolic factors*	Low peanut	High peanut	High peanut	Low peanut	*P* for trend
	(n = 284)	(n = 110)	(n = 242)	(n = 167)	
Abdominal obesity	1 (ref.)	1.17 (0.72, 1.89)	0.79 (0.52, 1.19)	0.60 (0.39, 0.93)	0.02
Hypertriglyceridemia	1 (ref.)	0.80 (0.45, 1.42)	0.66 (0.40, 1.08)	0.62 (0.36, 1.08)	0.06
Low HDL-cholesterol	1 (ref.)	0.83 (0.51, 1.33)	0.87 (0.57, 1.32)	1.11 (0.72, 1.70)	0.77
High blood pressure	1 (ref.)	0.85 (0.51, 1.41)	0.85 (0.55, 1.33)	0.84 (0.53, 1.33)	0.45
Hyperglycemia	1 (ref.)	1.07 (0.63, 1.84)	0.78 (0.48, 1.28)	0.69 (0.41, 1.15)	0.11

Multivariable logistic analysis was adjusted for age, gender, race, education (less than college, some college, college grad+), cigarette use (ever or never), alcohol use (ever or never), hours of sedentary time per day, energy, red meat, whole grain, and dairy intake.

Abdominal obesity: waist circumference ≥102 cm (≥40 inches) in men, ≥88 cm (≥35 inches) in women; hypertriglyceridemia: TG: ≥150 mg/dL (≥1.7 mmol/L) or drug treatment for elevated TG; low HDL-C: <40 mg/dL (<1.03 mmol/L) in men, <50 mg/dL (<1.3 mmol/L) in women, or drug treatment of reduced HDL-C; high blood pressure: BP: ≥130 mm Hg systolic BP, or ≥85 mm Hg diastolic BP, or drug treatment of hypertension; hyperglycemia: fasting glucose: ≥100 mg/dL or drug treatment for elevated glucose [4].

Multivariable-adjusted odds ratios of MetS and obesity according to nut intake are shown in Table 3. Compared to the low intake of nuts (LT/LP) group, the high nut consumers (HT/HP) had lower MetS, OR = 0.65 (95% CI, 0.72 – 1.00). With LT/LP intake as the reference, MetS was 23%, 35%, and 32% lower in LT/HP, HT/HP, and HT/LP consumers, respectively (*p* for trend = 0.056). When assessing tree nuts and peanuts separately, one serving of tree nuts per week (28 g/week) was significantly associated with 7% less MetS. Once per week consumption of tree nuts and total nuts was significantly associated with 3% and 2% reduction of MetS, respectively. Peanut consumption had no significant association with MetS.

**Table 3:** Multivariable-adjusted odds ratios (95% confidence interval) for prevalence of metabolic syndrome and obesity according to nut intake.

	Metabolic Syndrome		Obesity	
	OR	95% CI	OR	95% CI
*Type of nuts consumed*				
Low tree nut/Low peanut	1.0	Reference	1.0	Reference
Low tree nut/High peanut	0.77	(0.47, 1.28)	0.89	(0.53, 1.48)
High tree nut/High peanut	0.65	(0.42, 1.00)	0.63	(0.40, 0.99)
High tree nut/Low peanut	0.68	(0.43, 1.07)	0.54	(0.34, 0.88)
*P* for trend		0.056		0.006
*Frequency of nuts consumed*				
Tree nuts (1 time/week)*	0.97	(0.94, 0.99)	0.93	(0.89, 0.97)
Peanuts (1 time/week)†	1.01	(0.96, 1.06)	1.04	(0.98, 1.10)
Total nuts (1 time/week)	0.98	(0.96, 1.00)	0.97	(0.94, 0.99)
*Amount of nuts consumed*				
Tree nuts (1 serving/week)*	0.93	(0.88, 0.99)	0.90	(0.84, 0.97)
Peanuts (1 serving/week)†	1.05	(0.98, 1.12)	1.08	(1.00, 1.16)
Total nuts (1 serving/week)	0.98	(0.94, 1.01)	0.98	(0.94, 1.01)

Metabolic syndrome was defined according to the AHA/NHLBI diagnostic criteria [4].

Obesity: BMI ≥30 kg/m^2^ [25].

Adjusted for peanut intake, age, gender, race, education (less than college, some college,

college grad+), smoking (ever or never), alcohol use (ever or never), hours of sedentary

time per day, energy, red meat, whole grain, and dairy intake.

Adjusted for tree nut intake, age, gender, race, education (less than college, some college, college grad+), smoking (ever or never), alcohol use (ever or never), hours of sedentary time per day, energy intake, red meat, whole grain, and dairy intake.

Compared to LT/LP consumers, obesity was lower by 37% in HT/HP and 46% in HT/LP consumers (*p* for trend = 0.006). Further, obesity was significantly lower with intake of once per week of tree nuts and total nuts. When we considered the amount of tree nuts or peanuts consumed, obesity was significantly lower with one serving per week of tree nuts (OR = 0.90, 95% CI = 0.84 – 0.97), but higher with one serving per week of peanuts (OR = 1.08, 95% CI = 1.0 – 1.16).

## Discussion

The odds of metabolic syndrome and obesity were inversely related to the frequency, amount and the pattern of tree nuts and peanuts consumed in this North American population. Specifically, high consumers of tree nuts had the lowest prevalence of obesity and somewhat lower odds of MetS, which is consistent with our hypothesis.

We chose to categorize nut intake according to different levels of tree nut and peanut intake because in this population, as perhaps in others, consumption of tree nuts or peanuts is not exclusive of the other. Total nut intake among the highest tree nut consumers averaged 31.6 g, of which by weight 87% of the HT/LP and 53% of the HT/HP came from tree nuts. By comparison, nut consumers in the NHANES 1999–2004 study consumed on average 36.6 g total nuts, of which 91% came from tree nuts [17], the remaining presumably from peanuts. Nut consumers (≥2 servings/wk) from the Nurses' Health Study cohort had on average 28 g/d total nut intake of which approximately 79% came from tree nuts [30]. Interestingly, in the European Prospective Investigation into Cancer and Nutrition (EPIC) cohorts, the adjusted mean daily portion of tree nuts, peanuts and total nuts was 28.5 g, 46.5 g and 38.3 g, respectively, among male nut consumers. In females these were 23.1 g, 35.1 g, and 28.7 g, respectively [31]. It is possible that such differences in the nut intake pattern of these cohorts will also have varied impact on their metabolic and nutrient profiles.

Does the metabolic benefit come from total nuts or tree nuts? In cross-sectional analyses of data among adults from the NHANES 1999-2004 cohort, total nut consumption (tree nuts + peanuts) was associated with reduced prevalence of hypertension and low HDL-C, whereas tree nut intake was associated with lower prevalence of abdominal obesity, hyperglycemia, hypertension, low HDL-C, and MetS. In our cohort we found that high compared to low tree nut consumers had less abdominal obesity, and the odds of MetS tended to decrease with increasing tree nut intake, which is similar to the findings from a cross-sectional analysis of baseline data from the PREDIMED trial [32]. Lastly, investigators of a study in Finnish men reported that men with the highest compared to the lowest tertile of legume and nut intake had a 44% lower risk of MetS; however, the effect of nuts alone was not assessed [33].

Prospectively, total nut intake of ≥2 servings per week compared to <1 serving per week was associated with a 32% lower risk of MetS in a study of university graduates from Spain [18]. Our results suggest a possible 7% reduction in MetS for every 1 serving/week intake of tree nuts. Doubling this consumption could potentially reduce MetS risk by 14%. Additional evidence of the beneficial effect of tree nuts comes from the PREDIMED study, a randomized clinical trial to determine the efficacy of the Mediterranean diet (MedDiet) on the prevention of cardiovascular disease. In this clinical trial, the incidence rates for MetS were not different among the low-fat control diet, MedDiet + olive oil, or MedDiet + mixed nuts; however, reversal of MetS was observed in the MedDiet enriched with mixed tree nuts (15 g of walnuts, 7.5 g hazelnuts, and 7.5 g almonds) compared to the control diet [20]. Thus, while total nuts may have beneficial effects on some metabolic risk factors, findings from these cohorts suggest that the consumption of tree nuts may further prevent MetS. Nuts are excellent sources of polyunsaturated fatty acids, vitamin E, magnesium, and phytochemicals, properties which contribute to their favorable cardiometabolic effects [34].

Our finding of lower BMI among high tree nut consumers compared to low tree nut consumers is consistent with other reports. BMI has tended to be lower with increasing tree nut intake in both cross-sectional [16], [17] and prospective [30] studies. Lower risk of obesity among tree nut consumers compared to non-consumers of tree nuts was also observed in other cohorts [17], [32]. Also, a review of a few prospective studies showed that nut consumption was not associated with a higher risk of weight gain [35]. Results of short-term dietary intervention studies with nuts suggest that adding nuts to habitual diets of free-living individuals does not lead to any appreciable weight gain [14], [36]. Similarly, a recent meta-analysis evaluating thirty-one randomized nut trials concluded that diets enriched with nuts do not increase body weight, BMI or waist circumference compared to diets without nuts [15]. The lack of weight gain associated with nut consumption may be explained by a number of mechanistic hypotheses. For example, a substantial amount of energy provided by nuts can be compensated by a lower intake of other energy-dense foods [37] due to nutrient displacement [38], satiation, or satiety [39]. The satiation/satiety effects of nuts have been attributed to their high fiber and vegetable protein content, and the physical characteristics of nuts which require increased mastication [13]. Fiber may lead to delayed gastric emptying and reduced absorption; macronutrients may lead to the production of gastrointestinal hormones with satiating effects such as cholecystokinin; while increased mastication can result in fecal losses of macronutrients [36], [40], [41]. It is also possible that energy content from nuts is overestimated by 32% as determined by the Atwater factors [42].

Only two studies on nut intake and MetS have national representation of the US population, both of which were generated from NHANES 1999-2004 data [16], [17]. The AHS-2 Calibration Study participants, although not representative of the US population, are geographically spread throughout the US and Canada. Unique features of this cohort include the church recommendation to abstain from cigarettes and alcoholic beverages. Limited confounding due to very low cigarette and alcohol use results in greater statistical power and makes this cohort particularly suited to test the effect of diet on cardiometabolic risk factors. However, some limitations of this study are apparent. First, we recognize that errors associated with the use of a FFQ in assessing dietary intake may lead to misclassification of the exposure, which may underestimate true associations. Residual confounding as well as unmeasured and unknown factors may influence the association between tree nut and peanut intake and MetS or BMI. Although the cross-sectional design does not provide evidence of a temporal relationship between tree nut intake and MetS or BMI, our findings corroborate with those from other cross-sectional, prospective and intervention studies that tree nut intake is associated with reduced MetS and obesity.

In conclusion, tree nut consumption in this population has strong inverse association with obesity, and favorable though weak associations with abdominal obesity and MetS independent of demographic, lifestyle and other dietary factors. Tree nut consumption in particular may confer beneficial effects on metabolic risk factors.
